# Listeria monocytogenes Sepsis With Meningitis Complicating Biologic Immunosuppression in a Patient With Haemophagocytic Lymphohistiocytosis Secondary to Adult-Onset Still’s Disease: A Case Report

**DOI:** 10.7759/cureus.117297

**Published:** 2026-10-01

**Authors:** Haider Khan, Muhammad Navid Bin Mohamad Sithik, Amrita Gurung, Eranda L Ranasinghe Arachchi, Gary Meenagh

**Affiliations:** 1 General Internal Medicine, Southern Health and Social Care Trust, Craigavon, GBR; 2 Geriatrics, Southern Health and Social Care Trust, Craigavon, GBR; 3 Gastroenterology, Northern Health and Social Care Trust, Causeway, GBR; 4 Medicine, Health Education England, Cambridge, GBR; 5 Rheumatology, Northern Health and Social Care Trust, Antrim, GBR

**Keywords:** adult bacterial meningitis, adult-onset still’s disease (aosd), anakinra treatment, haemophagocytic lymphohistiocytosis (hlh), immunosuppression, listeria monocytogenes, listeriosis, opportunistic bacterial infection

## Abstract

Adult-onset Still’s disease (AOSD) and haemophagocytic lymphohistiocytosis (HLH) are treated with potent immunosuppression, including high-dose corticosteroids and biologic agents such as anakinra, which can result in an increased risk of opportunistic infections such as listeriosis (infection with *Listeria monocytogenes *(L. *monocytogenes*)). This case report describes a 34-year-old man on a background of AOSD receiving escalated doses of steroids and anakinra for a probable flare-up of HLH. Whilst inpatient, he developed high-grade pyrexia (39.3°C), acute confusion, and neurological deterioration, requiring admission to the ICU for intubation and ventilatory support. Investigations revealed blood cultures positive for *L. monocytogenes*, and lumbar puncture findings were consistent with acute bacterial meningitis. The patient clinically improved with targeted antimicrobial therapy and was successfully extubated, with subsequent normalisation of his inflammatory markers. He was discharged with a planned taper of his immunosuppressive therapy. This case emphasises the synergistic risk of infection associated with biologic therapy and high-dose corticosteroid use and the importance of maintaining a low threshold for considering listeriosis in immunocompromised patients, particularly in the presence of neurological deterioration.

## Introduction

Adult-onset Still’s disease (AOSD) is a rare autoinflammatory disorder characterised by inflammatory polyarthritis, quotidian fevers, and a salmon-pink maculopapular rash. It occurs at a rate of 0.16 to 0.4 cases per 100,000 individuals per year [1]. Haemophagocytic lymphohistiocytosis (HLH) is a hyperinflammatory syndrome resulting from dysregulated macrophage-lymphocyte interactions and cytokine hyperactivation. It can occur secondary to infection, malignancy, or autoimmune/autoinflammatory disease and may be difficult to recognise in patients with rheumatic disease [2,3]. AOSD can be complicated by secondary HLH, often referred to as macrophage activation syndrome (MAS) in rheumatological practice [4]. MAS is the term commonly used for secondary HLH occurring in association with rheumatological and autoimmune diseases such as AOSD.

Anakinra is a recombinant human IL-1 receptor antagonist used in AOSD and has increasing evidence in the treatment of secondary HLH/MAS [5]. Combination immunosuppression, particularly high-dose corticosteroids together with biologic therapy, increases the risk of serious and opportunistic infection [6]. *Listeria monocytogenes* (*L. monocytogenes*) infection has previously been reported in patients receiving tumour necrosis factor-alpha inhibitors, including infliximab, particularly where other immunosuppressive treatment was also used [7, 8]. Distinguishing HLH/MAS from superimposed infection may be challenging, as both can present with pyrexia, systemic inflammation, elevated inflammatory markers and hyperferritinaemia. Additionally, infection itself may precipitate or amplify secondary HLH/MAS, while escalation of immunosuppression to control the hyperinflammatory state may simultaneously increase susceptibility to infection.

*L. monocytogenes* is a Gram-positive facultative intracellular pathogen, usually acquired through contaminated food. It can cross the intestinal, blood-brain, and placental barriers [9]. Control of infection depends on cellular immunity, particularly T-cell and macrophage responses [10]. Although listeriosis is uncommon, it can cause serious invasive disease, including bacteraemia and meningitis, in patients with impaired immunity [11].

We report a case of culture-proven *L. monocytogenes* sepsis complicated by acute bacterial meningitis in a patient receiving high-dose corticosteroids and anakinra for AOSD-associated HLH. This case demonstrates the diagnostic and management challenges that can arise when severe infection develops during treatment of AOSD-associated HLH/MAS with high-dose corticosteroids and IL-1 blockade.

This case was previously presented as a poster at the British Society of Rheumatology annual conference 2026 and was published as an abstract poster in the conference booklet (DOI: 10.1093/rheumatology/keag121.045). 

## Case presentation

A 34-year-old man of Nigerian descent, working as an allied healthcare professional in a nursing home, presented to the emergency department (ED) with a three-week history of generalised non-pruritic urticarial rash, productive cough, rhinorrhoea, and sore throat consistent with a lower respiratory tract infection (LRTI). He also reported generalized bodyache with no joint swelling. Relevant social history included a recent relocation from London to Northern Ireland in February 2025; his most recent travel to Nigeria was in 2022. He denied tobacco use, alcohol consumption, intravenous drug use, known medical comorbidities, herbal medication use, freshwater swimming, animal contact, or relevant environmental exposures. 

Initial investigations revealed normocytic hypochromic anaemia (haemoglobin 93 g/L), elevated white cell count (WBC) of 11.7 × 10⁹/L and C-reactive protein (CRP) of 149 mg/L, and deranged liver function tests (LFTs) with alanine transaminase (ALT) 257 U/L, aspartate aminotransferase (AST) 211 U/L and gamma-glutamyl transferase (GGT) 168 U/L. The chest radiograph was unremarkable, and SARS-CoV-2 and influenza testing were negative. He was commenced on empirical antibiotics (amoxicillin 500 mg three times daily and clarithromycin 500 mg twice a day for treatment of the LRTI and was reviewed in the day assessment unit (DAU) four days later for low Hb cause. Relevant blood results were summarised in Table 1. 

**Table 1:** Quantitative and qualitative results across four key clinical timepoints. Reference intervals are those used by the Northern Health and Social Care Trust Clinical Pathology Laboratory for an adult male. The triglyceride reference interval assumes a 14-hour fast. ‘Not available’ indicates that no result was available for that time point. Abbreviations: ALT, alanine aminotransferase; AST, aspartate aminotransferase; CMV, cytomegalovirus; CRP, C-reactive protein; CSF, cerebrospinal fluid; HLH: haemophagocytic lymphohistiocytosis

Parameter, unit (reference interval)	Admission 1 Initial	Admission 2 HLH relapse	At *Listeria* diagnosis	Outpatient review three days after discharge
Quantitative results				
Haemoglobin (g/L; 130-170)	93	103	108	108
WBC (× 10⁹/L; 4-10)	11.7	6.8	17.7	6.2
C-reactive protein (CRP; mg/L; <5)	149	25	252	<1
Platelet count (× 10⁹/L; 150-410)	325	215	246	173
Ferritin (µg/L; 30.0-400)	2,893	53,708	23,349	2,886
Fibrinogen (g/L; 1.6-4.7)	8.39	2.55	7.7	2.27
Total bilirubin (µmol/L; 0-21)	19	106	115	15
ALT (U/L; 0-41)	257	2,763	1,155	119
AST (U/L; 0-40)	211	1,630	288	Not available
Triglyceride (mmol/L; 0.40-1.70)	1.95	2.05	2.18	2.11
CSF WBC (cells/µL)	Not available	Not available	560 (80% polymorphonuclear leukocytes; 20% lymphocytes)	Not available
CSF protein (g/L)	Not available	Not available	2.37	Not available
CSF glucose (mmol/L)	Not available	Not available	4.5	Not available
Maximum temperature (°C; reference interval not applicable)	39.6	36.1	39.3	Not available
Qualitative results				
Blood culture	Negative	Negative	Listeria monocytogenes	Not available
CMV testing	CMV PCR detected, very low load (<34.5 IU/mL);	CMV PCR not detected; IgG positive and IgM negative (past exposure)	Not available	Not available
CSF culture	Not available	Not available	Negative	Not available
CSF PCR	Not available	Not available	Negative	Not available

At the DAU review, his rash and respiratory symptoms had resolved; however, he had developed arthralgia of the left ankle, right knee and left wrist. He stated that all joints were affected simultaneously. On examination, the left wrist was swollen, warm, tender on palpation, and had a markedly reduced range of motion. His right knee was similarly tender without effusion. Repeat blood tests showed worsening anaemia (83 g/L), persistently elevated infection and inflammatory markers, along with a serum ferritin of 21,000 µg/L. He was admitted and commenced on piperacillin-tazobactam 4.5 g three times daily for infection of unknown source, as he had a temperature of 38.5°C along with raised infection markers. Comprehensive infectious, hepatic, autoimmune, and rheumatological screens were all negative, along with echocardiography and cross-sectional imaging (CT of the chest, abdomen, and pelvis), which revealed no abnormalities. Subsequently, a bone marrow biopsy was performed, which showed no evidence of haemophagocytosis.

Whilst in-patient, his ferritin and fibrinogen rose significantly to 52,881 µg/L and 8.39 g/L respectively. The H-Score was calculated at 162 (estimated probability of HLH approximately 40%-54%), raising suspicion for HLH. Given his symptoms (pyrexia for more than one week, polyarthritis, non-itchy skin rash) and laboratory investigations (deranged liver function tests, negative infective and autoimmune tests), AOSD was eventually diagnosed using the Yamaguchi criteria (two major and three minor) [12], and methylprednisolone and subcutaneous anakinra 300 mg daily were commenced. During in-patient care, low-level cytomegalovirus (CMV) viraemia was identified, but subsequent testing did not confirm any active infection. Following a positive clinical and biochemical response, he self-discharged while on subcutaneous anakinra 300 mg daily, oral prednisolone 60 mg daily, co-trimoxazole 480 mg three times weekly, omeprazole 20 mg daily, and vitamin D. He was enrolled in rheumatology outpatient follow-up.

At the planned DAU review following self-discharge, the patient reported symptomatic improvement. Prednisolone was reduced to 50 mg daily, and antihistamines were added for injection-site pruritus. At the six-week review, inflammatory markers were normal, and ferritin had improved to 2,940 µg/L. Anakinra was therefore reduced to 200 mg daily, and steroid tapering was continued as planned. Examination revealed non-pruritic, small follicular lesions on the back, consistent with a cutaneous manifestation of AOSD or a drug-related eruption. 

Two weeks after the last review, the patient presented with a one-week history of malaise, progressive polyarthralgia, generalised pruritus, jaundice, and dark urine. Clinical examination confirmed icterus. Investigations revealed severely deranged LFTs, and serum ferritin rose to 53,708 µg/L, indicating a significant HLH relapse. Other relevant laboratory results are presented in Table 1. He was re-admitted, and prednisolone was escalated to 60 mg daily while anakinra was restored to 300 mg daily. 

He was also investigated for malignancy with MRI, CT of the thorax, abdomen, and pelvis, tumour markers, and skin biopsy to detect intravascular lymphoma, all of which were normal. A liver biopsy performed during this admission demonstrated severe hepatitis with confluent necrosis. Additional evaluation for hepatitis aetiology included viral hepatitis serology, Epstein-Barr virus (EBV), repeat CMV testing (repeat CMV PCR was negative, consistent with past exposure), autoimmune liver antibodies, immunoglobulins, and alpha-1 antitrypsin, all of which were unremarkable. Co-trimoxazole, which had been continued since his last admission, was discontinued owing to concern for drug-induced liver injury (DILI), and his corticosteroid therapy was eventually escalated and converted to intravenous administration. Hyperglycaemia, likely steroid-induced, was difficult to control and required gliclazide and a subcutaneous insulin correction scale. A repeat bone marrow biopsy confirmed active haemophagocytosis with increased macrophage activity without clonal B cell proliferation (Figure 1). The H-Score was recalculated at 180 (estimated probability of HLH approximately 70%), confirming a hyperinflammatory state.

**Figure 1:**
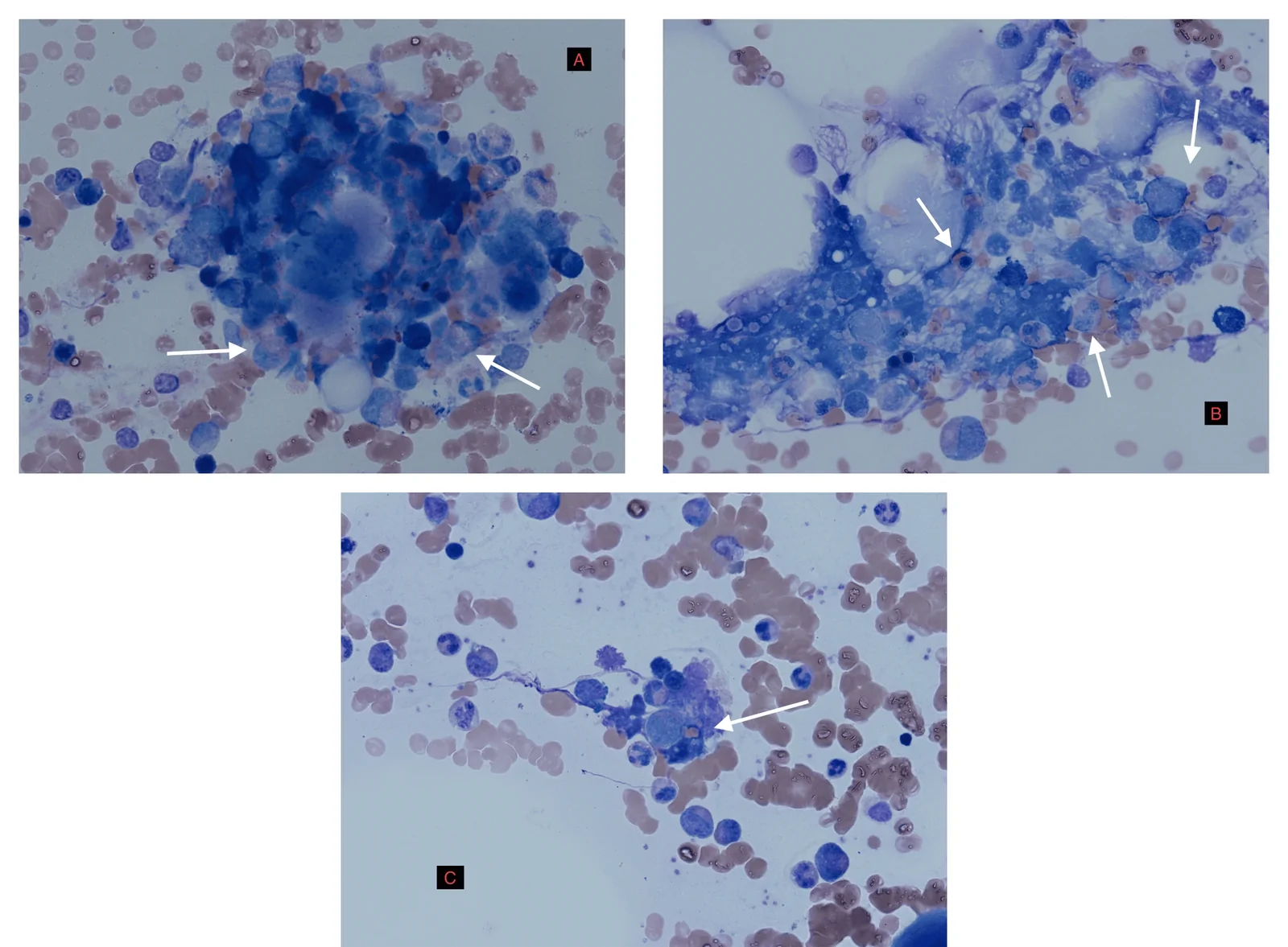
Bone marrow sample demonstrating haemophagocytosis. (A-C) Representative bone marrow images. White arrows indicate histiocyte/macrophages containing phagocytosed haematopoietic cells, consistent with haemophagocytosis.

However, during inpatient care, the patient developed pyrexia of 39.3°C accompanied by confusion, combativeness, and a reduced Glasgow Coma Scale Score (GCS) of 8/15. His clinical course deteriorated rapidly with progressive agitation, a reduced GCS, hyperglycaemia, and hyponatraemia, necessitating urgent ICU admission for intubation and mechanical ventilation. His inflammatory markers had also risen, and broad-spectrum antibiotic therapy with Tazocin 4.5 g three times daily was commenced, while a single dose of gentamicin 320 mg was administered whilst infectious investigations were undertaken. Blood cultures collected during this episode grew *L. monocytogenes*. Blood cultures were incubated and monitored using the BACT/ALERT® VIRTUO® automated blood culture system (bioMérieux Inc., Durham, NC, USA), and species identification was performed using VITEK® MS PRIME MALDI-TOF mass spectrometry (bioMérieux). Antimicrobial susceptibility testing was performed using minimum inhibitory concentration methodology with Liofilchem MIC Test Strips (Liofilchem S.r.l., Terumo, Italy) on Mueller-Hinton agar supplemented with 5% defibrinated horse blood and 20 mg/L β-NAD, with results interpreted according to European Committee on Antimicrobial Susceptibility Testing (EUCAST) breakpoints for *Listeria *[13]. The antimicrobial panel tested comprised penicillin, amoxicillin, and co-trimoxazole. The isolate was subsequently referred to the UK Health Security Agency Bacteriology Reference Department at Colindale for reference characterisation, including *Listeria *species identification, serotyping and typing of *L. monocytogenes* by whole-genome sequencing and single-nucleotide polymorphism analysis.

Lumbar puncture yielded straw-coloured cerebrospinal fluid (CSF), with a white cell count of 560 cells/µL comprising 80% polymorphonuclear leukocytes and 20% lymphocytes, and a red cell count of 110 cells/µL. CSF protein was elevated at 2.37 g/L, and CSF glucose was 4.5 mmol/L. The corresponding serum glucose was 15.4 mmol/L, giving a CSF-to-serum glucose ratio of approximately 0.29. No organisms were seen on CSF microscopy, and CSF culture demonstrated no growth after 48 hours of incubation. Bacterial meningitis PCR testing was negative for *Neisseria meningitidis*, *Streptococcus pneumoniae*, and *Haemophilus influenzae*. In the context of concurrent *L. monocytogenes* bacteraemia, the neutrophil-predominant CSF pleocytosis, elevated protein, and reduced CSF-to-serum glucose ratio were consistent with acute bacterial meningitis. The diagnosis of listerial sepsis with meningitis was confirmed. Key quantitative and qualitative findings across the initial presentation, HLH relapse, culture-confirmed listeriosis, and early outpatient follow-up are summarised in Table 1. 

A focused dietary history confirmed consumption of non-heat-treated food products during his inpatient stay. These dietary exposures, combined with his immunosuppressed state (steroids and anakinra), significantly increased the risk of developing listeriosis.

Targeted antimicrobial therapy was initiated with intravenous amoxicillin 2 g four times daily for a total of 21 days, combined with gentamicin 5 mg/kg divided into three doses daily for the first seven days. Anakinra was reduced to 200 mg daily. The patient demonstrated a sustained clinical response, with gradual recovery of consciousness allowing extubation and subsequent normalisation of inflammatory markers and LFTs over the following two weeks. He was discharged in stable condition on anakinra 200 mg daily and prednisolone 20 mg daily with planned tapering. Outpatient follow-up was uneventful. The clinical course, investigations, and management are summarised chronologically in Table 2. 

**Table 2:** Chronological summary of the clinical course, investigations and management Dates are presented chronologically from the patient's initial emergency department presentation. Abbreviations: AOSD, adult-onset Still's disease; CNS, central nervous system; CSF, cerebrospinal fluid; DAU, day assessment unit; ED, emergency department; GCS, Glasgow Coma Scale; HLH, haemophagocytic lymphohistiocytosis; ICU, intensive care unit; MAS, macrophage activation syndrome; PCR, polymerase chain reaction.

Time-point	Clinical course	Key investigations/findings	Management/outcome
Day 1: Initial ED presentation	Three-week history of generalised non-pruritic rash with respiratory symptoms and systemic inflammatory features.	Anaemia, raised inflammatory markers and deranged liver enzymes; chest radiograph unremarkable; SARS-CoV-2 and influenza testing negative.	Empirical oral antibiotics commenced for presumed lower respiratory tract infection; DAU review arranged to further investigate cause of anaemia.
Day 5: DAU review/first admission	Respiratory symptoms and rash had improved, but new polyarthralgia/polyarthritis and pyrexia prompted hospital admission.	Marked hyperferritinaemia; extensive infectious, hepatic, autoimmune and rheumatological investigations unrevealing. Initial bone marrow biopsy showed no haemophagocytosis; H-score raised suspicion of HLH/MAS.	Empirical IV piperacillin-tazobactam commenced. Rheumatology assessment supported AOSD-associated HLH/MAS.
Days 7-8: Treatment initiation	Treatment for the hyperinflammatory state was initiated during the first admission.	Clinical and laboratory findings considered consistent with AOSD-associated HLH/MAS.	IV methylprednisolone commenced on 2 April for three days; subcutaneous anakinra commenced on 3 April.
Day 15: Self-discharge	Clinical and biochemical improvement after treatment.	Inflammatory markers and ferritin improving suggested response to treatment.	Patient self-discharged on anakinra 300 mg daily, prednisolone 60 mg daily and supportive/prophylactic medication; rheumatology follow-up arranged.
Day 18: First post-discharge DAU review	Reported symptomatic improvement.	No new concerning clinical findings documented.	Continued with anakinra 300 mg daily and prednisolone 60 mg daily
Day 64: Rheumatology review	Continued clinical and biochemical improvement.	Inflammatory markers normalised and ferritin substantially improved.	Anakinra reduced to 200 mg daily; planned corticosteroid taper continued.
Day 85: Second admission/HLH-MAS relapse	Re-presented with malaise, progressive polyarthralgia, generalised pruritus, jaundice and dark urine.	Marked hyperferritinaemia and severe hepatitis. Malignancy, viral and autoimmune investigations were unrevealing. Repeat bone marrow biopsy during the admission demonstrated haemophagocytosis; H-score increased.	Anakinra increased to 300 mg daily, and prednisolone increased to 60 mg daily; Co-trimoxazole discontinued because of suspected drug-induced liver injury.
Day 99: New febrile episode	Developed a new high-grade temperature during the second admission.	Blood cultures obtained during the febrile episode.	Infectious work-up initiated and antimicrobial therapy escalated while results were awaited.
Day 101: Listeriosis diagnosed/ICU admission	Acute confusion, agitation and reduced GCS with rapid neurological deterioration; required intubation and ICU admission.	Blood cultures collected in the first week of July reported positive for *Listeria monocytogenes*.	Targeted IV amoxicillin and gentamicin commenced; mechanical ventilation and ICU supportive care provided. Anakinra subsequently reduced.
Day 101-102: Lumbar puncture	Neurological deterioration investigated for CNS infection.	CSF demonstrated neutrophil-predominant pleocytosis, elevated protein and reduced CSF-to-serum glucose ratio, consistent with acute bacterial meningitis. No organisms seen; culture showed no growth after 48 hours. Bacterial meningitis PCR was negative for *Neisseria meningitidis*, *Streptococcus pneumoniae* and *Haemophilus influenzae*.	Targeted antimicrobial treatment continued.
Day 103: Early recovery	Neurological and clinical improvement.	Improving clinical status following antimicrobial therapy and ICU management.	Successfully extubated; antimicrobial treatment continued.
Day 122: Hospital discharge	Continued clinical recovery with improvement in neurological and systemic features.	Inflammatory markers and liver function tests improved.	Discharged in stable condition after completing/continuing the planned antimicrobial course; discharged on reduced immunosuppression with planned taper and follow-up.
Day 126: Post-discharge follow-up	Early outpatient review was uneventful.	Ongoing clinical and biochemical improvement.	Continued rheumatology follow-up and immunosuppression taper.

## Discussion

Central nervous system (CNS) infection is a well-recognised severe manifestation of listeriosis, particularly in patients with underlying immunosuppression [14]. *L. monocytogenes* is a facultative intracellular pathogen and a major cause of foodborne infection in immunocompromised individuals. Following ingestion, it can evade intracellular killing through production of listeriolysin O, a pore-forming toxin that allows escape from the phagosome into the cytosol [15]. It can then spread from cell to cell by actin-based motility, mediated by ActA, and by phospholipase activity [9]. Entry into the CNS may occur through the choroid plexus, brain microvasculature, or infected phagocytes, resulting in meningitis, meningoencephalitis, rhombencephalitis, or brain abscess [16]. This explains the patient’s presentation with acute bacterial meningitis, reduced GCS score, and rapid neurological deterioration. Therefore, in any immunosuppressed patient presenting with fever and neurological deterioration, there should be a high index of suspicion for infection, including listeriosis, even where the clinical picture may initially resemble an inflammatory flare.

In this patient, the H-Score increased from 162 to 180 during relapse, and repeat bone marrow examination confirmed haemophagocytosis. This justified escalation to high-dose methylprednisolone and anakinra. However, infection is also a recognised trigger of secondary HLH [2,3]. It is therefore possible that evolving *L. monocytogenes* infection contributed to, or amplified, the hyperinflammatory state rather than occurring solely as a consequence of intensified immunosuppression. Blood cultures obtained during the HLH relapse were negative, with *L. monocytogenes* only isolated during the subsequent episode of pyrexia and neurological deterioration. Therefore, a causal relationship between occult listeriosis and the preceding HLH relapse cannot be established.

Anakinra is increasingly used in AOSD-associated HLH/MAS that is refractory to corticosteroid therapy [5]. Experimental work in murine listeriosis has shown that endogenous IL-1 is important for neutrophil recruitment and macrophage activation [17]. This provides a possible biological explanation for susceptibility to *Listeria *during IL-1 blockade; however, it does not prove that anakinra caused the infection in this patient. The use of high-dose corticosteroids was another important factor that increased infection risk by affecting adaptive immunity and macrophage function [6]. The contribution of co-trimoxazole discontinuation should also be considered. Trimethoprim-sulfamethoxazole has good CSF penetration and has been used in the treatment of *Listeria *meningitis [18]. A similar case has been reported in a patient with steroid-dependent AOSD receiving infliximab, in whom co-trimoxazole was discontinued because of suspected DILI before *Listeria *meningoencephalitis developed [19]. In the present case, co-trimoxazole was also stopped because of concern regarding DILI. However, the temporal relationship alone does not prove that prophylactic-dose co-trimoxazole prevented listeriosis.

This case also highlights the importance of a focused dietary history and infection-prevention counselling in immunocompromised inpatients. The patient had consumed non-heat-treated food products during his inpatient stay. People receiving oral steroids, biologics, or other immunosuppressive treatment are recognised by the UK Health Security Agency as being at increased risk of invasive listeriosis and should avoid certain chilled ready-to-eat foods [20]. Prompt antimicrobial treatment is important because *L. monocytogenes* is inherently resistant to third-generation cephalosporins. Ampicillin or amoxicillin, often combined with gentamicin, remains standard treatment for invasive listeriosis [21]. UK meningitis guidance also emphasises early recognition, appropriate investigation, and prompt empirical treatment in patients with suspected bacterial meningitis [22]. In this case, targeted treatment with intravenous amoxicillin for 21 days and gentamicin for the first seven days resulted in gradual recovery and subsequent normalisation of inflammatory markers and liver function tests. Reducing anakinra rather than completely withdrawing immunosuppression was a pragmatic approach, balancing infection control with the risk of further HLH relapse.

## Conclusions

This case describes L. monocytogenes bacteraemia with concurrent acute bacterial meningitis in a young immunocompromised patient with AOSD-associated HLH/MAS. High-dose corticosteroid therapy and IL-1 blockade with anakinra represented important immunosuppressive factors that may have increased susceptibility to invasive infection. Although co-trimoxazole had been discontinued, the temporal association does not establish that prophylactic-dose co-trimoxazole would have prevented listeriosis. The development of unexplained pyrexia and neurological deterioration in an immunocompromised patient should prompt consideration of invasive listeriosis alongside other causes of bacterial meningitis. Prompt microbiological investigation and appropriate antimicrobial therapy are essential, while modification of immunosuppression should balance infection control against the risk of recurrent hyperinflammation. Increased clinical awareness of opportunistic infection remains an important component of care in patients receiving significant immunosuppression.
